# On Looking the Age of Consent

**Published:** 1921-04-16

**Authors:** 


### THE EDITOR'S LETTER-BOX.

**Correspondence on all subjects is invited, but we cannot in any way be responsible for the opinions expressed by our correspondents, who must give their name and address as a guarantee of good faith, but not necessarily for publication. Correspondents are reminded that brevity of style and conciseness of statement greatly facilitate early insertion.**

### On Looking the Age of Consent.

*To the Editor of* The Hospital.

Sir,—The hush-a-bye-baby policy advocated, and the diversity of opinions expressed by the various noble Lords in the course of a recent debate on raising the age of consent to seventeen, might imply how little the average person is capable of appreciating those affairs that affect him much. From his tree-top he reviews that Lord Phillimore believes the taking away of the defence of fair cause to imagine that the girl is over a certain age will dispense with reasonable defence in the case of all young people. Further, the young people do not really commit a crime, because the girl is only getting what she asks for. Apart from the truth that a girl's charm is increased by her uncertain age, perhaps no unhappier example of the obtuseness of the law could possibly be given than the fore-going—a blown wind which (if reported correctly) is all that is necessary to set many cradles rocking. For if it is a fact that no crime has been really committed, how can certain beliefs dispose of necessary defence? Whilst our Judges of Assize are increasingly bending the bough by handing out hard labour to this one and that for offences against the moral laws of the country, there is at least one judge who considers that it is not a crime for a girl to ask and a boy (of eighteen or nineteen) to give, always provided she looks her age. But would it be well generally to accept the tip-and-run ruling of his Lordship? Methinks it might raise the ire of full many a bishop. Its unsteadying application to the general public would, I feel sure, result in the premature downfall of babies, cradles, and all.—Yours truly,

April 6. Momus.

